# Antiviral mechanism of carvacrol on HSV-2 infectivity through inhibition of RIP3-mediated programmed cell necrosis pathway and ubiquitin-proteasome system in BSC-1 cells

**DOI:** 10.1186/s12879-020-05556-9

**Published:** 2020-11-11

**Authors:** Li Wang, Dan Wang, Xingan Wu, Rui Xu, Yunlan Li

**Affiliations:** 1 The First Affiliated Hospital of Xi’an Medical University, Xi’an, China; 2 Department of Scientific Research, the Second Affiliated Hospital of Xi’ an Medical University, Xi’an, China; 3 Department of Pathogenic Microorganism, School of Preclinical Medicine, Air Force Medical University, Xi’an, China; 4 School of Pharmaceutical Science, Shanxi Medical University, No. 36, Xin Jian South Road, Taiyuan, 030001 China

**Keywords:** Carvacrol, Herpes simplex virus-2 (HSV-2), Antiviral activity, Programmed cell necrosis, Ubiquitin-proteasome

## Abstract

**Background:**

Carvacrol, as the major components of aromatic plants used for treating human skin diseases including origanum, Satureja, thymus, and coridothymus species, presented a kind of antiviral activity. To explore the mechanisms of carvacrol against herpes simplex virus (HSV) in vitro.

**Method:**

The BSC-1 cells model of HSV infection was established, and from the two aspects of viral replication level and cell death pathway, the antiviral effects of carvacrol on HSV infected cells were also evaluated by plaque assay under the three modes including prevention, treatment, and direct inactivation.

**Results:**

In the three ways, the half-maximal effective concentration (EC_50_) of 2% true carvacrol solution on HSV-2 infected cells were severally 0.43, 0.19 and 0.51 mmol/L, and the corresponding therapeutic index (TI) were 4.02, 9.11 and 3.39, respectively. It’s the opposite of the increased levels caused by HSV-2 infection, that both the expressions at the transcription genes and protein levels of virus own replication key factors (including ICP4, ICP27, VP16, gB, and UL30) and cytokines (including RIP3, TNF-α, and MLKL) of infected cells treated with carvacrol were dose-dependently inhibited. Besides, HSV-2 infection can cause the decrease of intracellular protein ubiquitination level, and carvacrol can reverse the ubiquitination decrease level caused by HSV-2 infection.

**Conclusion:**

Carvacrol exhibits significant antiviral activity by inhibiting the HSV-2 proliferation process and HSV-2-induced TNF-α increasing levels, decreasing RIP3 and MLKL protein expressions through the intracellular RIP3-mediated programmed cell necrosis pathway. In addition, carvacrol also may exhibit anti-HSV-2 activity by reversing the ubiquitination decrease level caused by HSV-2 infection on the ubiquitin-proteasome system, which provides insights into the molecular mechanism.

**Supplementary Information:**

The online version contains supplementary material available at 10.1186/s12879-020-05556-9.

## Background

Herpes simplex virus (HSV) is enveloped, linear and double-stranded DNA virus, which belongs to the family *herpesviridae*, genus *alphaherpesvirinae*. It is one of the most common pathogenic agents in humans and divided into two types: HSV-1 and HSV-2 [1]. There is increasing awareness of the importance of skin infection disease caused by HSV infection. Several of emerging cases were found continuously in recent years [2, 3], which indicated an upward trend in HSV incidence. Extensive evidence proved that HSV viruses could typically cause severe afflictions, mainly because of the generation of genital lesions and severe infections like life-threatening encephalitis and disseminated infections in neonates [4–6]. The HSV is also associated with potentially fatal viral stromal keratitis, an ocular disease, which is a leading cause of cornea-derived blindness in developed countries [7]. Currently, the approved primary anti-HSV therapeutic drugs are acyclovir (ACV) and its derivatives, which interfere with viral DNA synthesis to reduce viral replication and transmission. But all mentioned nucleoside analogs drugs, including ACV, ganciclovir and penciclovir, were oriented on the same molecular mechanisms of action that hinders viral DNA synthesis by competitively inhibiting viral DNA polymerase or adding itself to viral DNA, and the multi-drug resistant HSV viral strains were starting to show up more and more with heavy use of nucleoside analogs agents. Besides, the disadvantages of their narrow antiviral spectrum and high costs gradually also aggravated people’s living burden [8–10], extremely in America [11]. For these reasons, there is a need for the development of novel antiherpes drugs which are safe and preferably inexpensive with limiting the primary infection and supporting further treatment.

A large number of herbs and aromatic plants are frequently used for treating human skin diseases, especially from the family of *Lamiaceae* including origanum, satureja, thymus, and coridothymus species [12]. Carvacrol, a monoterpene phenol that is also known as 2-methyl-5-(1- methyl ethyl)-phenol, is one of the significant components of oregano essential oils, and presents a wide diversity of biological activities, such as antiviral [13–15], anticancer [12], antimicrobial [16], antioxidant and anti-inflammatory [17, 18]. Besides, carvacrol also has been identified as a natural, economical food preservative. Currently, the carvacrol-related health products, including 60 soft gels and 60 vegetarian capsules, are available for antioxidant treatments on the market. The relevant literature have also indicated that carvacrol has an safety and tolerability effect on healthy volunteers through a phaseIclinical trial and possible therapeutic effect on asthmatic patients through a phase II clinical trial in recent years [19, 20]. Carvacrol could exert antiviral activity by preventing the death of cells infected with HSV, but the specific mechanism of it against HSV virus has not been reported up to now [21, 22]. As a new alternative energy, carvacrol provides a new possibility for the development of HSV treatment and preventive health care drugs with advantages of great source, safety, low toxicity, and nature.

So the purpose of this paper was to explore the antiviral activity of carvacrol against HSV in vitro by plaque assay. The possible mechanisms of carvacrol’s antiviral effect on HSV-2 infected BSC-1 cells were studied from two aspects of viral replication level and cell death pathway through molecular biological techniques, which can provide adequate theoretical supports for the discovery of new antiviral drugs and alternative energy.

## Methods

### Carvacrol, cells, virus strains and major reagents

Carvacrol (Oregano oil; Purity: 99.8%) and 2% carvacrol true solution, prepared by dissolving 2 mL carvacrol with 33% sulfur-β-paste in distilled water at 100 mL, were kindly provided by prof. S. W from the air force medical university in China. Vero cells and HSV laboratory standard virus strain (HSV-1-F strain and HSV-2-G strain) were kindly gifted by prof. X. A from the air force medical university. BSC-1 cells were kindly donated by prof. Z. Q from Wuhan University. Vero and BSC-1 cells were incubated under Dulbecco-modified eagle’s medium (DMEM, high glucose) with 10% fetal bovine serum (FBS) at 37 °C in the atmosphere containing 5% CO_2_. HSV strains were grown for 3 ~ 4 days on cells in an atmosphere of 5% CO_2_ at 37, and the virus stock solution was stored at − 80 °C until use. Whereafter, the plaque assay [23] was performed to determinate viral multiplicity of infection (MOI) on cells. Of which, high glucose DMEM medium, FBS and cell counting CCK8 kits were purchased from Shanghai sangon biological engineering co. Ltd. The following antibodies were used: anti-ICP4 polyclonal antibody (Abcam; ab96432), anti-ICP27 monoclonal antibody (Abcam; ab31631), anti-VP16 monoclonal antibody (Abcam; ab110226), anti-gB monoclonal antibody (Abcam; ab6506), anti-Caspase-3 antibody (Abcam; ab 90,437), anti-Ub antibody (Abcam; ab7780), anti-RIP3 (Abcam; ab56164), anti-MLKL antibody (Abcam; ab184718), anti-Caspase-1 (Proteintech; 22,915–1-AP), anti-TNF-α (Proteintech; 60,291–1-lg). Goat anti-rabbit infrared secondary antibody (IR Dye800CW, 926–32,211) and goat anti-mouse infrared secondary (IR Dye680RD; 926–68,070) were purchased from American LICOR biosciences. Secondary antibody binding to Alexa Fluor 488 or Cy3 was purchased from Xi’an Zhuangzhi biotechnology co.Ltd. Fast 1000 total RNA rapid extraction kit, 5 × PrimeScript RT Master Mix (TakaRa), RNase Free dH_2_O (TakaRa) and TB GreenTM Premix Ex TaqTM II (TakaRa) were obtained from Xi’an kehao biological engineering co. Ltd.

### Screening of HSV-infected cells and viral titer determination

Plaque assays were performed with a monolayer culture of Vero and BSC-1 cells in 6-well plates. The cell monolayer was infected with HSV-1 and HSV-2 virus stock at multiple dilutions (10^− 2^, 10^− 3^, 10^− 4^, 10^− 5^, 10^− 6^, 10^–7),^ respectively, and incubated at 37 °C with 5% CO_2_ incubator (Thermo HERACELL 150i, America) for 2 h. Cell monolayer infected without HSV-2 virus was used as the blank control. The infected cell monolayer was then overlaid with an overlapping solution containing 2% carboxymethyl -cellulose sodium salt. After 3 ~ 4 days, the cell monolayer was washed three times with PBS and stained with 1% crystal violet solution. Plaques were counted and plaque formation units (PFUs / mL) were calculated as^−^*x / (n* × v) × d, where^−^*x*, *n*, v, and d refer to the average numbers of plaques, repetitive holes numbers, viral load and dilution factor, respectively.

### Cell morphology changes

The HSV-1 and HSV-2 virus solution were respectively added to BSC-1 cell monolayers for 2 h to allow viral attachment. After 2 h’ incubation, the virus solution was replaced with the maintenance media (DMEM supplemented with 2% FBS). The cellular state was observed and photographed under an optical microscope (20 × magnification) using visible light (OLYMPUS-CKX31, Japan).

### Cytotoxicity determination of carvacrol on BSC-1 cells

The cytotoxicity of carvacrol was determinated by CCK-8 assay [24]. BSC-1 cells were seeded in 96-well plates and cultured in 10% DMEM for 24 h at 37 °C in an atmosphere containing 5% CO_2_. The medium was then removed and carvacrol (with dose of 1, 0.8, 0.7, 0.6, 0.5, 0.4, 0.3, 0.2, 0.1, 0.05, 0.025 and 0 mmol/L) and 2% carvacrol real solution (with dose of 4, 3.5, 3, 2.5, 2, 1.8, 1.6, 1.4, 1.2, 1, 0.8 and 0 mmol/L) were severally added to individual wells of BSC-1 cells in plates with 3 wells in parallel for each dose and the plates were incubated for 24 h. Cells treated without the carvacrol were used as a control. After that, the supernatant medium of each well was replaced with 100 μL DMEM, and 10 μL CCK-8 solution was added to the cells, and cells were cultured for 4 h avoiding light. The absorbance (A) of each well was measured at 450 nm using BioTek synergy2 microplate reader. The cell viability was calculated using the following formula: Cell viability (%) = (*A*_s_ - *A*_b)_ / (*A*_c_ - A_b)_ × 100%, where *A*_s_ and *A*_c_ refer to the absorbance in the presence and absence of carvacrol, respectively, and *A*_b_ stands for the blank control. Subsequently, the half-maximal inhibitory concentration (IC_50_) of carvacrol and 2% carvacrol real solution on BSC-1 was automatically calculated using Bliss principle according to the cell viability values obtained above.

### Screening on the antiviral activity of carvacrol against HSV-1 and HSV-2

For treatment assay, the BSC-1 cell monolayer was firstly incubated with HSV-1/2 viruses (MOI = 0.05) at 37 °C for 2 h to allow viral attachment. Following 2 h’ incubation, the carvacrol with different concentrations (1, 0.5, 0.25, 0.125, 0.0625, 0 mmol/L) were added to the infected cells for 24 h. After that, the supernatant was replaced with the overlapping solution containing 2% carboxymethyl - cellulose sodium salt and cells were continued to incubate at 37 °C for 3 ~ 4 days. The infected cell monolayer was then strained by plaque assay. The cell monolayer without treatment of carvacrol was used as a virus control group. Plaques were counted and the antiviral activity was calculated as [(*V*_C_ - *V*_D_) / *V*_C_] × 100%, where *V*_D_ and *V*_C_ refer to the plaques’ number in the presence and absence of carvacrol.

For pre-treatment assay, BSC-1 cells monolayer was first treated with carvacrol at the same as above concentrations for 24 h at 37 °C before HSV-1/2 (at MOI = 0.05) with plaque assay. For viral direct inactivation assay, the HSV-1/2 virus supernatant and carvacrol at the same indicated concentrations were added to the cell culture, simultaneously. The infected cell monolayer was co-cultured with virus and carvacrol for 24 h at 37 °C using plaque assay.

### Antiviral activity of carvacrol and 2% carvacrol true solution against HSV-2

The antiviral activities of carvacrol and 2% true carvacrol solution against HSV-2 were severally observed by the above three assays of pre-treatment, treatment, and viral direct inactivation according to the above formula, respectively. The 50% effective concentration (EC_50_) of them on HSV-2 infected BSC-1 cells was then calculated and the rapeutic indexes (TI) were further calculated in three modes using the following formula: TI = IC_50_ / EC_50_.

The time-of-addition assay was performed as described in the literature [25]. Briefly, carvacrol at 0.5 mmol/L concentration was added into cell monolayer at the different time point (0, 3, 6, 9, 12, 24 h) after virus infection (MOI = 0.00025, that is 50PFUs / well). At 24 h post-infection (p.i.), the solution was replaced with the overlapping medium containing 2% carboxymethyl-cellulose sodium salt. The cells were continued to incubate for 3 ~ 4 days and strained for plaque assay.

The time-of-removal assay as described [26]. Briefly, carvacrol at 0.5 mmol/L concentration was added into cell monolayer after virus adsorption. At the different post-infection time, the solution was removed, and the overlapping medium was added into cell monolayer, subsequently. Following 3 ~ 4 days’ incubation, the cell monolayer was strained by plaque assay.

### Virus release assay

The BSC-1 cell monolayer was infected with HSV-2 (at MOI = 0.03) for 2 h. Different concentrations of carvacrol (1, 0.5, 0.25, 0.125, 0.0625, 0 mmol/L) were added into cells after viral attachment. After 24 h of incubation at 37 °C with 5% CO_2_, the supernatant and cell pellet were collected, respectively. The cell pellet was subjected to freeze-thaw cycles of three times before titration. Virus titers of supernatant and cell pellet were determined by plaque assay, and the virus release rate of carvacrol at different concentrations against HSV-2 was also calculated using the formula: the virus release rate (%) = *T*_ex_ / (*T*_ex_ + *T*_in_) × 100%, where *T*_ex_ and *T*_in_ represent extracellular and intracellular virus titer, respectively.

### Immunofluorescence assay

For the effect analysis of carvacrol on the expression sites of HSV-2 ICP27 and gB protein, BSC-1 cells were grown on the glass coverslips at a density of 5.0 × 10^4^ cells / well and cultured in DMEM with 10% FBS overnight. The indicated concentration of carvacrol was added to the cell monolayer after HSV-2 infection (at MOI = 2). After 24 h incubation, cells were washed with PBS and fixed with 4% paraformaldehyde for 20 min, permeabilized with 0.5% Triton X-100, and then blocked with 3% bovine serum albumin (BSA) for 1 h at 37 °C. The coverslips were then incubated for 1 h at 37 °Cwith the indicated primary antibody at a dilution of 1: 1000 [including anti-ICP27, anti-gB and anti-RIP3]. Subsequently, alexa fluor 488 or cy3 conjugated goat anti-mouse IgG secondary antibody at a dilution of 1: 200 was added into cells for 1 h’ incubation avoiding light. And cells were washed and stained with 4′,6-diamidino-2-phenylindole (DAPI) at room temperature for 10 min. The coverslips were captured with the fluorescence microscope.

### Western blot analysis

For the HSV-2 pivotal proteins and cytokines proteins analysis, a total of 2.5 × 10^5^ cells were seeded in 6-well plate before HSV-2 infection. Infected cells treated with different post-infection time and a different dose of carvacrol were lysed with RIPA lysis buffer for 30 min on the ice. Besides, normal BSC-1 cells, as a control, were also lysed after 24 h’ incubation. The protein supernatants at different treatment groups were collected and quantified using BCA protein quantification kit. After being denatured by boiling, equivalent amounts of protein (40 μg) were separated on SDS-PAGE, transferred to 0.45 μm polyvinylidene difluoride (PVDF, 619534, Sangon Biotech, Shanghai) membranes, and blocked with 5% non-fat milk in TBST for 1 h. Then, the membrane was incubated with primary antibody at a dilution of 1:1000 [including anti-ICP4, anti-ICP27, anti-VP16, anti-gB, anti-Caspase-3, anti-Ub, anti-RIP3, anti-MLKL, anti-Caspase-1, anti-TNF-α) at 4°Covernight, followed by infrared fluorescence secondary antibodies with a dilution of 1:10000 (IR Dye800CW goat anti-rabbit antibody, Dye 680RD goat anti-mouse antibody, LICOR, USA). Membrane were visualized using the Odyssey infrared imaging system (model: 9120, LICOR, USA).

### Q-PCR analysis

For the mRNA expression of HSV-2 key genes analysis, total RNA of the infected cells treated with different post-infection time and the different dose of carvacrol was extracted with trizol according to the procedure of fast 1000 total RNA rapid extraction kit and reverse-transcribed into cDNA with 5 × PrimeScript RT Master Mix. Gene expressions were detected by Q-PCR analysis with SYBR Green enzyme amplification program, including preincubation at 95 °C for 30 s and 45 amplification cycles with degeneration at 95 °C for 10 s and anneal at 60 °C for 31 s. The melting curves of each gene was analyzed by Roche instrument operation instructions. The products of ICP4, ICP27, VP16, gB, and GAPDH were separated on 2% agarose gel electrophoresis, and amplification fragments of each gene were observed and photographed. The sequence of primers used in PCR analysis was shown in Table 1.

**Table 1:** The sequence lists of the primers used in Q-PCR analysis

Genes	Primer sequence (5′-3′)		Length (bp)
*ICP4*	Forward	GATGGGGTGGCTCCAGAAC	103
	Reverse	AGATGAAGGAGCTGCTGTTGC	
*ICP27*	Forward	CCCTTTCTGCAGTGCTACCT	95
	Reverse	CCTTAATGTCCGACAGGCGT	
*VP16*	Forward	AATGTGGTTTAGCTCCCGCA	103
	Reverse	CCAGTTGGCGTGTCTGTTTC	
gB	Forward	CCATGACCAAGTGGCAGGAG	103
	Reverse	AGGTTGGTGGTGAAGGTGGTC	
*UL-30*	Forward	AGATCAAGGTGAACGGGATGG	141
	Reverse	GTCGCGGTAGCTCAGATCCTT	
*GAPDH*	Forward	CAAGAAGGTGGTGAAGCAGGC	171
	Reverse	CATACCAGGAAATGAGCTTGAC	
*RIP-3*	Forward	CATAGGAAGTGGGGCTACGAT	95
	Reverse	AATTCGTTATCCAGACTTGCCAT	
*MLKL*	Forward	AGGAGGCTAATGGGGAGATAGA	70
	Reverse	TGGCTTGCTGTTAGAAACCTG	
*TNF-α*	Forward	GAGGCCAAGCCCTGGTATG	91
	Reverse	CGGGCCGATTGATCTCAGC	
*TNF-R1*	Forward	TCACCGCTTCAGAAAACCACC	96
	Reverse	GGTCCACTGTGCAAGAAGAGA	

### Statistical analysis

All experiments were repeated three times. All calculations, including IC_50_, EC_50_, *t*-test, were performed using GraphPad Prism 7.0 software (GraphPad company, USA). Student’s *t*-test was used to compare the treatment groups with the control and determine the statistical significance. Significant differences were indicated by *p*-value (*, *p* < 0.05; **, *p* < 0.01; ***, *p* < 0.001).

## Results

### Cell model establishment of the HSV-infected BSC-1 cells and viral titer

It’s well known that Vero cells have been used as one of the HSV susceptible cell lines. To clarify whether BSC-1 cells can be infected with the HSV virus, the viral titer of both HSV-1 and HSV-2 were compared on the different cell lines: Vero and BSC-1 cells.

**Fig. 1:**
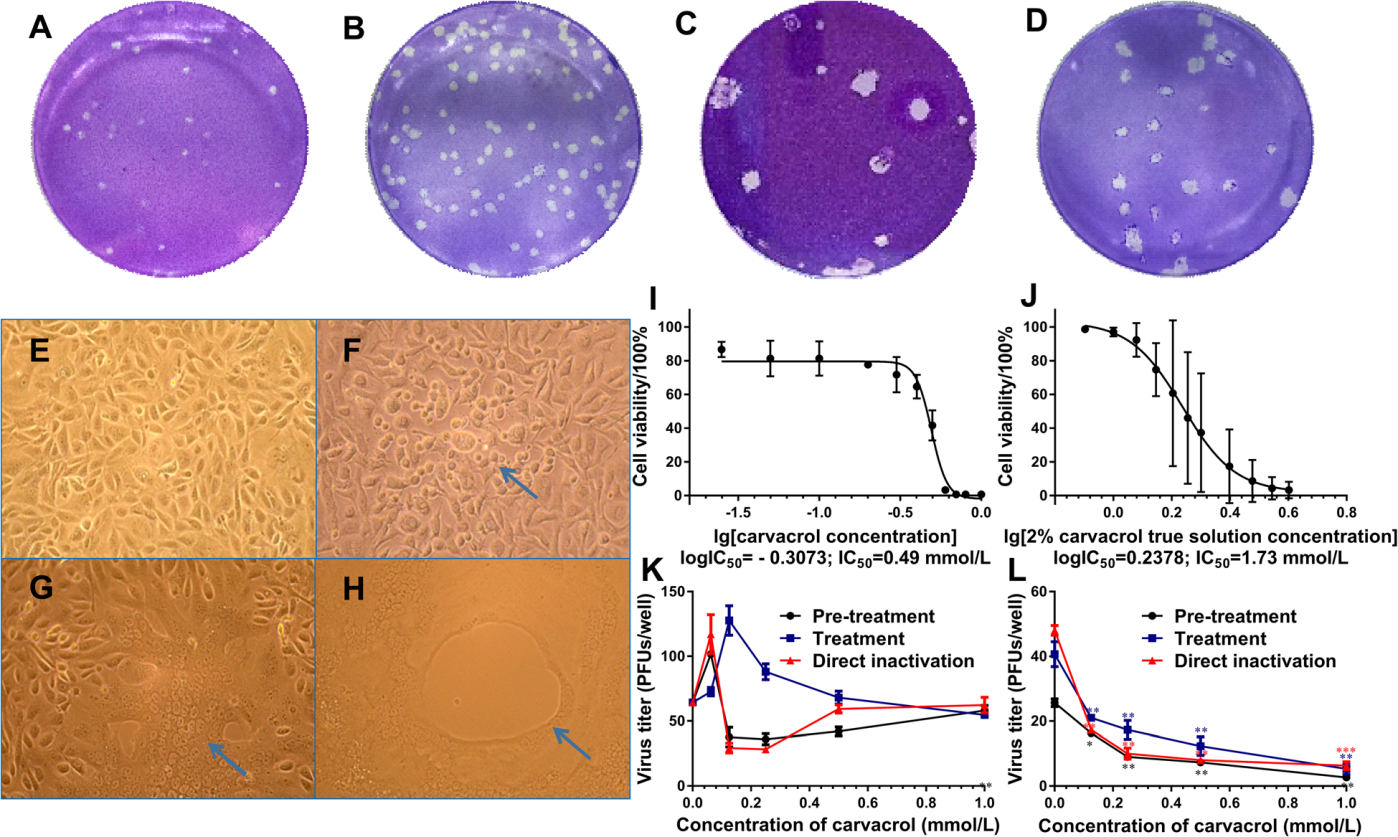
The viral titer determination of HSV-1 and HSV-2 on different cell lines. **a** Vero cells infected with HSV-1 at 10^− 3^ dilution; (**b**) BSC-1 cells infected with HSV-1 at 10^− 3^ dilution; (**c**) Vero cells infected with HSV-2 at 10^− 6^ dilution; (**d**) BSC-1 cells infected with HSV-2 at 10^− 6^ dilution. The morphological changes of BSC-1 cells infected HSV-1 or HSV-2 (200 ×). (**e**) The normal BSC-1 cells; (**f**) BSC-1 cells infected with HSV-1 for 24 h; (**g**) BSC-1 cells infected with HSV-2 for 24 h; (**h**) BSC-1 cells infected with HSV-2 for 30 h. The cellular survival curves of BSC-1 cells treated with carvacrol and 2% true carvacrol solution. **i** carvacrol; (**j**) 2% true carvacrol solution. The antiviral activities of carvacrol against HSV-1/HSV-2. (K) HSV-1; (I) HSV-2. *, *p* < 0.05; **, *p* < 0.01; ***, *p* < 0.001

As shown in Fig. 1, the plaque formation numbers of HSV-1 at 10^− 3^ dilutions on Vero and BSC-1 cells (Fig. 1a and b) were twenty-nine and ninety-five, respectively. At 10^− 6^ dilutions of HSV-2 (Fig. 1c and d), the plauqe numbers on Vero and BSC-1 were eleven and twenty-one, respectively. So the viral titers of HSV-1 and HSV-2 on Vero cells were 3.2 × 10^4^ and 1.2 × 10^7^ PFUs/mL, while the viral titers on BSC-1 cells were 1.1 × 10^5^ PFUs/mL and 2.3 × 10^7^ PFUs/mL, respectively. It was observed (from Fig. 1) that the viral infection titers of HSV-1 and HSV-2 on BSC-1 cells were higher than that of Vero. Therefore, the BSC-1 cell model of HSV infection was established in this study.

### Morphological changes of BSC-1 cells

The cellular morphology can be seen from Fig. 1. Normal BSC-1 cells were arranged tightly with uniform tadpole-like morphology and intact cytomembrane, and the cell membranes were clearly visible with a strong refractive index (Fig. 1e). However, BSC-1 cells became round, swollen and partially fell off after HSV-1 infection for 24 h (Fig. 1f). The cell membranes of HSV-2 infected cells were blurred, and multiple cells were gradually merged to form a multinucleated giant cell (Fig. 1g), which shed off to appear vacuoles with HSV-2 post-infection time rising to 30 h (Fig. 1h).

### Cytotoxicity assessment of carvacrol and 2% carvacrol real solution on BSC-1 cells

To evaluate the usabilities of carvacrol and 2% carvacrol real solution, their cytotoxicity was determined using CCK-8 cell counting kit. Cellular survival curves presented in Fig. 1 clearly shown that the logIC_50_ value of carvacrol (Fig. 1i) and 2% carvacrol real solution (Fig. 1j) on BSC-1 cells were − 0.3073 and 0.2378, so the corresponding IC50 value is calculated as 0.49 and 1.73 mmol/L, respectively. So the cytotoxicity of 2% carvacrol real solution was lower than that of carvacrol. So, 2% carvacrol real solution was used for further studying the mechanism of Carvacrol on HSV infectivity.

### Comparison of antiviral activities of carvacrol between HSV-1 and HSV-2

In this study, plaque assay was used to compare the anti-HSV-1 and anti-HSV-2 viral activities of carvacrol in three ways of pre-treatment, treatment, and direct inactivation. As shown in Fig. 1k, carvacrol at different doses showed inconsistent and unstable inhibitory effect on the virus titer of HSV-1. However, it can dose-dependently reduce the virus titer of HSV-2 with a consistent downward trend and a significant difference in the above three ways (Fig. 1l). Therefore, the antiviral activity and mechanism of carvacrol against HSV-2 in vitro were selected for further study.

### The anti-HSV-2 activity of carvacrol in vitro under pre-treatment, treatment and direct inactivation modes of HSV-2 infection

**Fig. 2:**
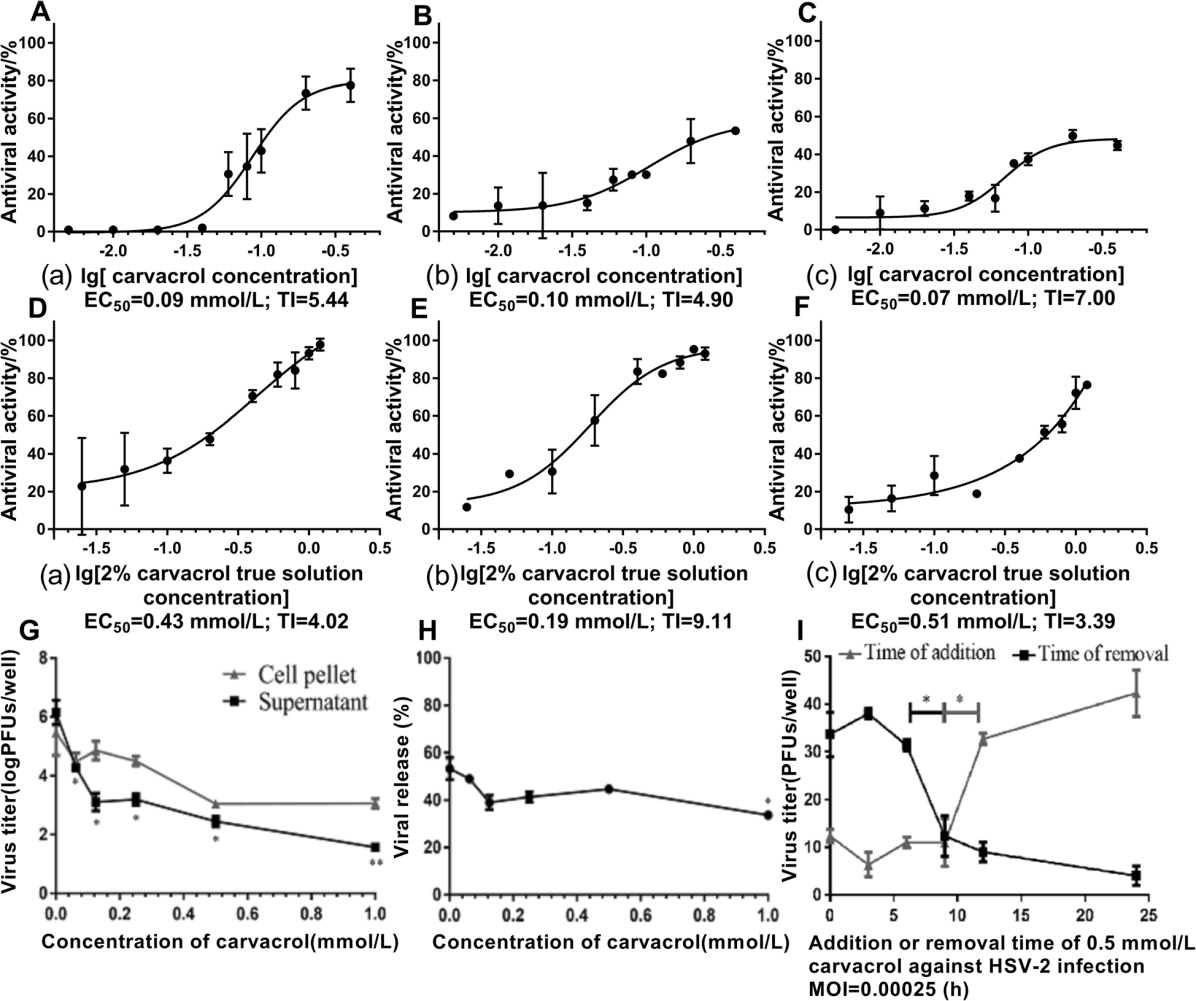
The dose-response curves of carvacrol and 2% true carvacrol solution against HSV-2 in different modes. (A, B, C) carvacrol; (D, E, F) 2% true carvacrol solution; (a) pre-treatment; (b) treatment; (c) direct inactivation. The effects of carvacrol on HSV-2 virus release. (G) the viral titers of culture supernatant and intracellular virus; (H) the viral release rate of HSV-2. (I) The effect of delay addition and early removal of 0.5 mmol/L carvacrol on viral titer (MOI = 0.00025). *, *p*<0.05; **, *p* < 0.01

In this study, the dose-response curves of carvacrol and 2% true carvacrol solution against HSV-2 were obtained in Fig. 2, respectively. The corresponding EC_50_ and TI were calculated by the formula above. Under three modes of pre-treatment (Fig. 2A(a)), treatment (Fig. 2B(b)) and viral direct inactivation (Fig. 2C(c)), the EC_50_ value of carvacrol on HSV-2 infected cells were 0.09, 0.10 and 0.07 mmol/L, and the corresponding TI were 5.44, 4.90 and 7.00, respectively. However, the EC_50_ value of 2% true carvacrol solution against HSV-2 in the three ways (Fig. 2D(a), 2E(b) and 2F(c)) were 0.43, 0.19 and 0.51 mmol/L, and its corresponding TI were 4.02, 9.11 and 3.39, respectively. So both the carvacrol and 2% true carvacrol solution can provide variously antiviral effects for pre-treatment, treatment and direct inactivation modes of HSV-2 infection.

### Effect of carvacrol on virus release

To determine whether carvacrol could have an effect on virus release, the extracellular and intracellular viral titer in the presence and absence of carvacrol were compared. As shown in Fig. 2g, when 0.0625 mmol/L carvacrol was applied to HSV-2 infected cells, the viral titer of culture supernatant was significantly decreased and the inhibition rate of carvacrol against HSV-2 was 30% (4.28 logPFUs/mL vs 6.15 logPFUs/mL), while the intracellular virus titer can be slightly attenuated (4.48 logPFUs /mL vs 5.46 logPFUs/mL; the inhibition rate was only 18%). As shown in Fig. 2h, the HSV-2 virus release rate was gradually decreased with 1 mmol/L carvacrol. According to the formula of viral release rate, the viral release rate of HSV-2 was significantly reduced to 33.67 ± 2.05% at 1 mmol/L carvacrol.

### The effect time point of carvacrol on HSV-2 replication

The time-of-addition and time-of-removal assays were performed to determine the antiviral activity of carvacrol for HSV-2 at different replication stage.

As shown in Fig. 2i, the HSV-2 titer was 12 PFUs/well when carvacrol was added into cell monolayer immediately after HSV-2 infection (that is 0 point of addition, cells were treated with carvacrol 24 h), while the HSV-2 titer was 34 PFUs/well when carvacrol was immediately removed on cell monolayer treated with HSV-2 infection and carvacrol (that is 0 point of removal, cells were only treated with carvacrol 0 h). Compared with the addition of carvacrol (0.5 mmol/L) at 12 h for infected cells (HSV-2 MOI = 0.00025), The addition of carvacrol at 9 h can significantly reduce HSV-2 virus titer (33 PFUs/well, 12 h vs 11 PFUs/well, 9 h). The removal of carvacrol at 9 h resulted in a significant decrease for HSV-2 infection titer compared to 6 h (12 PFUs/well, 9 h vs 31 PFUs/well, 6 h), and the viral titer was gradually decreased as the carvacrol’s removal time was prolonged. These data suggested that the antiviral activity of carvacrol was related to the time of its addition or removal after a viral infection, and it can significantly affect HSV-2 replication between 6 h and 12 h post-infection time.

### Effects of carvacrol on the expression sites of ICP27 and gB protein in BSC-1 cells infected with HSV-2

Numerous evidence has shown that HSV immediate early (IE) genes play an important role in subsequent viral early protein expression. There are five IE genes: ICP0, ICP4, ICP22, ICP27, and ICP47. Of which, ICP27 is a multifunctional regulatory protein required for viral replication and could activate other early or late viral genes expression [27, 28]. The glycoprotein B (gB) on the HSV is a late-stage protein, which can promote viral adhesion for cells and accelerate the fusion of infected cells. As visible by 4′,6-diamidino-2-phenylindole (DAPI) staining, cells infected with HSV-2 exhibit distinct cytopathic effect with the character of nucleus aggregation.

Meanwhile, through immuno-fluorescence staining, the ICP27 and gB protein expression of HSV-2 were mainly located in the nucleus (green fluorescence, Supplement Fig 1A) and cytoplasm (red fluorescence, Supplement Fig 1B) of infected cells, respectively. And as shown in Supplement Fig. 1, the expression levels of both ICP27 and gB were negatively correlated with the dose of carvacrol.

### Carvacrol significantly attenuates HSV-2 replication at molecular expression levels

At previous experiments, it was shown that carvacrol has good anti-HSV-2 activity on infected cells. Here, real-time quantitative PCR (Q-PCR) and western blot were used to detect the molecular expression levels of HSV-2 virus-associated factors, including ICP4, ICP27, VP16, gB and UL30 of infected cells treated with different post-infection time and different doses of carvacrol.

Firstly, PCR electrophoresis and melting curves analysis were used to demonstrate the specificity and feasibility of designed primers in the corresponding gene. As shown in Supplement Fig 1C, agarose gel electrophoresis results of ICP4, ICP27, VP16, gB, and UL30 gene indicated that the amplification fragment of each gene was complete and single without specific bands appearing. And the melting curves of each gene presented a single peak (Supplement Fig. 1D-H ), which indicated that there was no primer dimer.

**Fig. 3:**
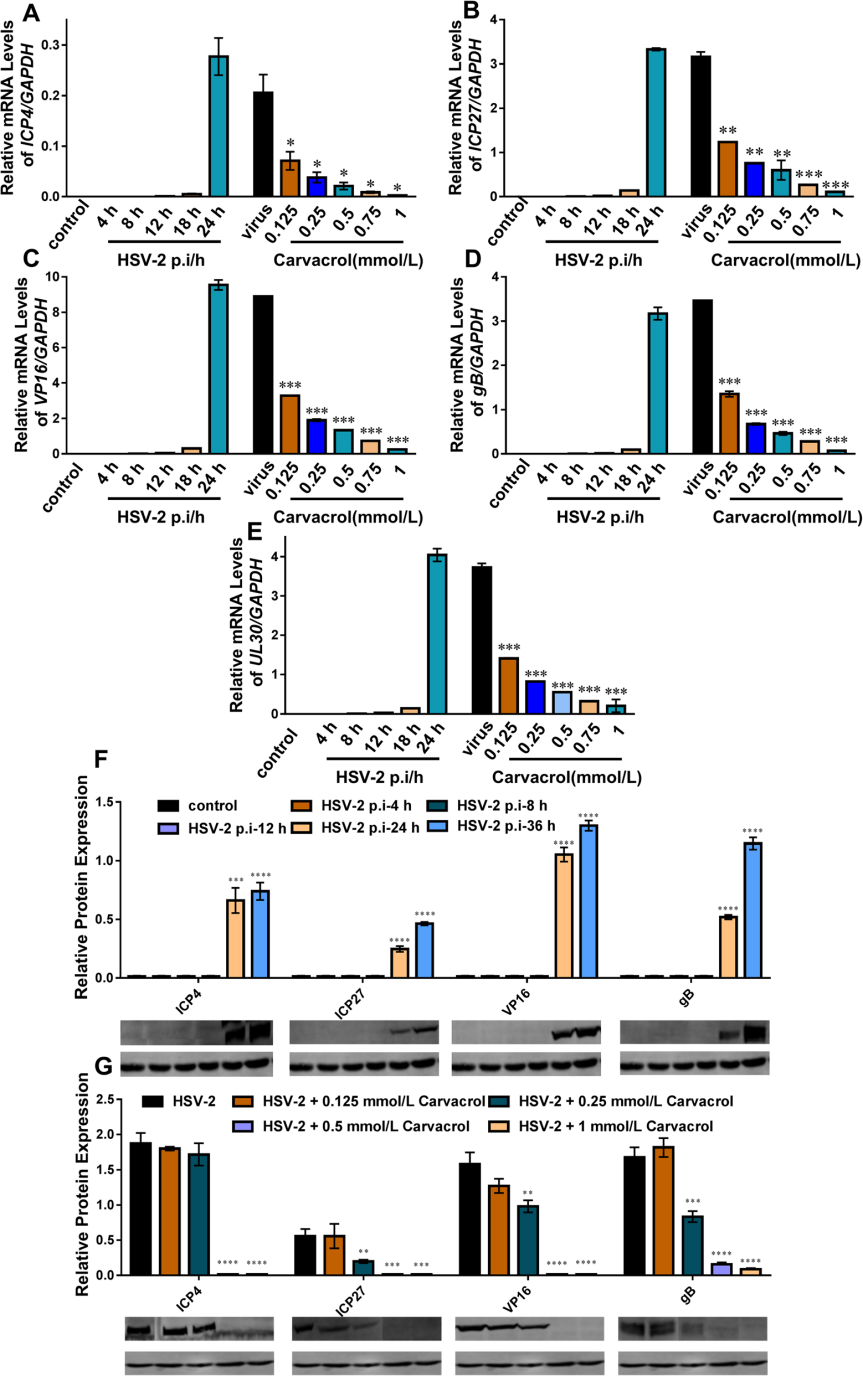
The molecular expression levels of each factor in cells treated with different post-infection time and different doses of carvacrol. **a**, **b**, **c**, **d**, and E represented mRNA levels (**a**. ICP4; **b**. ICP27; **c**.VP16; **d**. gB; **e**. UL30); **f** and **g** represented protein levels (**f**. the different HSV-2 post- infection time; **g**. the different concentration of carvacrol. *, *p* < 0.05; **, *p* < 0.01; ***, *p* < 0.001

Besides, the expressions of each gene (such as HSV-2 ICP4, ICP27, VP16, gB and UL30, and so on) at the levels of transcription genes (Fig. 3a-e) and protein (Fig. 3f, g) were gradually increased with the prolongation of HSV-2 infection time. But, these factors’ expressions levels of infected HSV-2 cells treated carvacrol for 24 h were significantly decreased in a dose-dependent manner (Fig. 3). Therefore, carvacrol may be play an antiviral activity by inhibiting the synthesis of HSV-2 key replicator genes.

### Relationship between HSV-2 infection and RIP3-mediated programmed cell necrosis

**Fig. 4:**
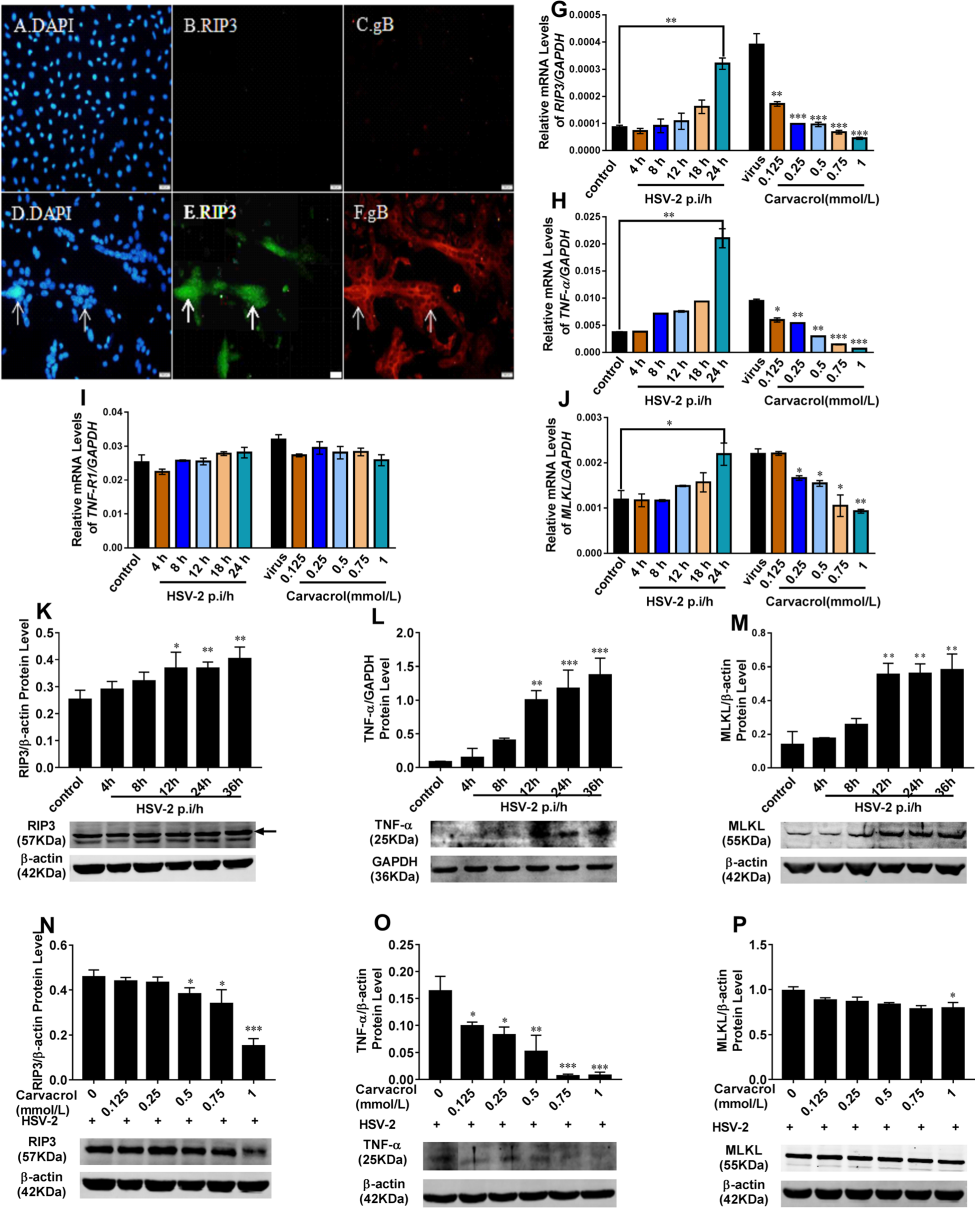
Immunofluorescence staining results of gB and RIP3 proteins in normal and infected cells. **a**, **b**, **c**: the normal cells; **d**, **e**, **f**: the infected cells. **g**-**p**:The gene and protein expression levels of RIP3, TNF-α, and MLKL in cells treated with the different post-infection time of HSV-2 and different carvacrol concentrations

Compared with the normal cell group (Fig. 4a, b, c), the expression level of HSV-2 gB protein in the lesion fusion site of infected cell was higher (red fluorescence, Fig. 4f), and the RIP3 protein expression can be also seen at the same site by immunofluorescence staining (green fluorescence, Fig. 4e), so HSV-2 may be initiate intracellular RIP3-mediated pro-grammed cell necrosis pathway, when HSV-2 infection induced multiple cell fusion to form polynuclear giant cells.

### Carvacrol showed a good anti-HSV 2 activity by inhibiting RIP3-mediated cell necrosis pathway

Firstly, it’s observed that the gene expression level of RIP3 in HSV-2 infected cells was time-dependently increased by Q-PCR (Fig. 4g-j).

Compared with the control group, the gene expression level of RIP3 at HSV-2 post-infection 24 h was significantly increased by 3.73 ± 0.05 times, then, its expression treated with carvacrol was significantly decreased with the dose rising (Fig. 4g). Expected, western blot results (Fig. 4k) showed that RIP3 protein expression level was positively correlated with HSV-2 post-infection time. And the relative protein expression of RIP3 was increased by 1.47 ± 0.17 times at HSV-2 post-infection 12 h (*p*<0.05). However, its expression increased level caused by HSV-2 infection was significantly inhibited (*p*<0.05) at 0.5 mmol/L carvacrol (Fig. 4n).

Besides, the relative molecular expressions level of upstream factors (TNF-α, TNF-R1) and downstream factors (MLKL) in RIP3-mediated programmed cell necrosis pathway were further evaluated at infected cells treated with the different post-infection time of HSV-2 and different carvacrol concentrations by Q-PCR and western blot assays. As shown in Fig. 4, with the prolongation of HSV-2 infection time, both the gene (Fig. 4h and j) and protein expressions (Fig. 4l and m) levels of TNF-α and MLKL were increased, but the expressions of TNF-R1 were not affected (Fig. 4i). At 24 h post-infection time, the transcriptional gene expression levels of TNF-α and MLKL were significantly increased by 5.65 ± 0.24 and 1.90 ± 0.38 times (*p*<0.01; *p*<0.05), respectively. Their protein expression was also significantly elevated, compared with cells untreated with HSV-2 infection. However, when the HSV-2 infected cells were treated with different doses of carvacrol for 24 h, the gene (Fig. 4h and j) and protein (Fig. 4o and p) expressions levels of TNF-α and MLKL were decreased to some extent, but the expressions of TNF-R1 were not affected compared with the virus control group (Fig. 4i). Of which, the gene expressions of TNF-α and MLKL in infected cells treated with 0.25 mmol/L carvacrol were significantly reduced to 0.57 ± 0.02 and 0.76 ± 0.04 times of the viral group (Fig. 4h and j), respectively. And the protein expression of TNF-α in infected cells treated with 0.125 mmol/L carvacrol was also decreased to 0.62 ± 0.09 times with a significant difference (Fig. 4o). Until the concentration of carvacrol rising to 1 mmol/L, the protein expression of MLKL was significantly weakened (Fig. 4p).

Through synthesis analysis on transcriptional gene and protein levels of RIP3, TNF-α, MLKL, and TNF-R1, HSV-2 infection might be initiate intracellular RIP3-mediated cell necrosis pathway by inducing intracellular TNF-α expression, activating RIP3 protein and promoting MLKL expression. When carvacrol was applied on infected cells, it may exhibit anti-HSV-2 activity by inhibiting HSV-2-induced TNF-α increased levels, decreasing RIP3 protein activity and weakening MLKL protein expression.

### Carvacrol exerts antiviral activity through affecting intracellular ubiquitin-proteasome degradation system

**Fig. 5:**
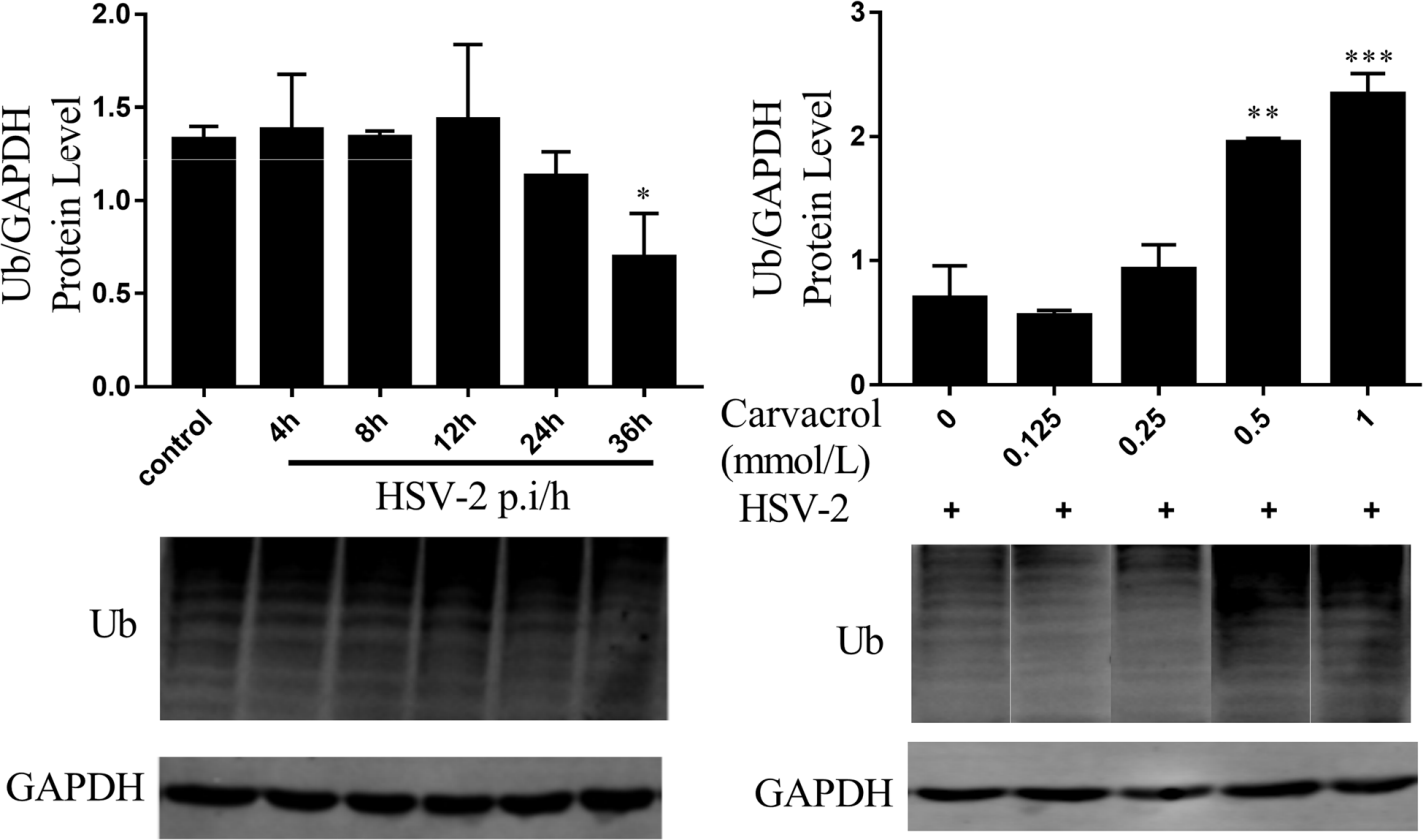
Effects of ubiquitination protein in cells treated with different post-infection time of HSV-2 and different carvacrol concentrations. *, *p* < 0.05; **, *p* < 0.01; ***, *p* < 0.001

The ubiquitin-proteasome system is another type of protein degradation pathway used to maintain homeostasis in the body. The virus could complete its life cycle by regulating the ubiquitination (Ub) of relevant proteins. As shown in Fig. 5, the Ub level of proteins in infected cells was reduced to 47.16 ± 0.17% at HSV-2 post-infection 36 h compared with the normal group (0.69 ± 0.19 vs 1.33 ± 0.06). On the contrary, when carvacrol at 0.5 mmol/L was applied to infected cells, the Ub level of the large molecular weight protein was increased to 3.03 ± 0.74 times of the virus control group, and there was a significant difference (*p* < 0.01). Therefore, HSV-2 infection could cause a decrease of intracellular protein ubiquitination level, and carvacrol can reverse the ubiquitination decrease caused by HSV-2 infection.

## Discussion

HSV proliferation can be accomplished by multiple processes of its adsorption, drilling, shelling, biosynthesis, assembly and release [29–31]. In the natural environment, the main host of HSV infection is human, but it has a full susceptibility to infect cells in the laboratory environment. Previous literatures [32, 33] have shown that HSV-2 infection models in vitro can be established on various cell lines, such as human embryonic lung fibroblasts (MRC-5), human endometrial adenocarcinoma cells (HEC-1-A), human cervical cancer cells (Hela), human lung cancer cells (A549), African green monkey kidney cells (Vero), and milk hamster kidney cells (BHK). Among them, Vero cells are classic cell lines for virus amplification. The growth of Vero cell is rapid and density-dependent, which might easily lead to cell stack growth. The state of cell growth is inconvenient for the exploration of the possible mechanism of carvacrol against HSV. Hence, it is necessary to find an alternative susceptible cell line for HSV infection. BSC-1 is a monkey kidney cell line, but it has a slow proliferation, and isn’t easy to stack growth, which can eliminate some interference factors for the study of the antiviral mechanism of carvacrol against HSV-1/2. In this study, the plaque assay was used to screen infected cell lines of HSV, and the results showed that BSC-1 cells could be infected with HSV, and the infection efficiency on the BSC-1 cells is higher than that of Vero cells. Therefore, BSC-1 cells could be used as a susceptible cell line for HSV infection.

In the three ways of pre-treatment, treatment and direct inactivation virus, both carvacrol and 2% true carvacrol solution could exert an individual antiviral activity on the infected HSV-2 cells by plaque assay, and their antiviral activities depend on the dosage form of carvacrol. Based on the analysis of EC_50_ and TI, 2% true carvacrol solution had an EC_50_ value higher than that of carvacrol on HSV-2 infected cells under the three modes. However, since the IC_50_ value of the former was higher than the latter, the TI of 2% true carvacrol solution was less than carvacrol in the two ways of pre-treatment and direct inactivation virus. So the safety of carvacrol is higher than that of the 2% true carvacrol solution in both modes, which makes it possible to develop carvacrol as preventive health products in the future. In the way of treatment, the TI value of 2% true carvacrol solution was higher than carvacrol, so the former was safer than the latter, which provided a theoretical basis for the development and utilization of 2% true carvacrol solution as a liquors.

HSV-2 is a relatively complex virus particle consisting of three parts: capsid, cortex, and envelope. And the surface of the envelope contains multiple protrusions. Rapidly, HSV-2 proliferates, with a replication cycle of approximately 8 ~ 16 h [1]. During replication of the virus, the HSV-2 gene can be divided into immediate early genes, early genes, and late genes according to the chronological order of gene transcription and translation [34]. There are five IE genes: ICP0, ICP4, ICP22, ICP27, and ICP47. Of which, ICP4 is an critical regulatory protein in HSV-2 replication, and ICP4 and ICP27 were used as immediate early regulatory proteins to initiate transcription and translation of early genes, which play an essential role in viral replication and cell growth [34–36]. The HSV virus capsid contains many capsid protein VP16, which entered the cellular nucleus and bound to the viral DNA to activate the expression of the early viral gene after the virus invaded the cells [33]. The gB on the HSV capsule envelope is a late-stage protein, which can promote viral adhesion for cells and accelerate the fusion of infected cells. Besides, the protein encoded by the HSV-2 UL30 gene is one of the subunits on the DNA polymerase and is critical for viral DNA synthesis [37]. It was found by plaque assay that carvacrol could decrease the HSV-2 virus titer; immunofluorescence staining results indicated that carvacrol could attenuate the expression of HSV-2 ICP27 and gB protein on spatial epitopes; Q-PCR results indicated carvacrol could inhibit the replication of HSV-2 virus at the transcription gene level. The western blot assay indicated that the carvacrol inhibited the vital proteins expressions of ICP4, ICP27, VP16 and gB in HSV-2 replication from the linear degree of the protein. According to the above comprehensive data, the antiviral activity of carvacrol on infected cells is closely related to the replication process of HSV-2.

It is known that the protein encoded by HSV-2 UL30 gene is one of the subunits of viral DNA polymerase and participates in the viral replication process [37]. Therefore, a small part of the experiments in this study was performed to detect the effect of carvacrol on the expression of HSV-2 UL30 gene and to elucidate further whether carvacrol might inhibit the replication of viral DNA by inhibiting UL30 subunit on DNA polymerase. The results (Fig. 3e) showed that the expression of UL30 gene was gradually increased with the HSV-2 post-infection time rising. When carvacrol was administered to infected cells, the expression level of UL30 was significantly inhibited in a dose-dependent manner, which means that the anti-HSV-2 activity of carvacrol may be related to the replication process of viral DNA by interfering with UL30 subunit on DNA polymerase.

To a certain extent, the HSV viruses could escape cellular defense mechanisms, such as HSV-2, which can establish latent infection in the human body. In the cell infection model test in vitro, it was observed that the infected cells were gradually fell off and died with the prolongation of HSV-2 post-infection time. At present, cell death pathways mainly include autophagy, apoptosis, pyroptosis, and RIP3-mediated programmed cell necrosis pathways. And it has been reported that viral infection is closely related to the above cell death pathways [38, 39].

**Fig. 6:**
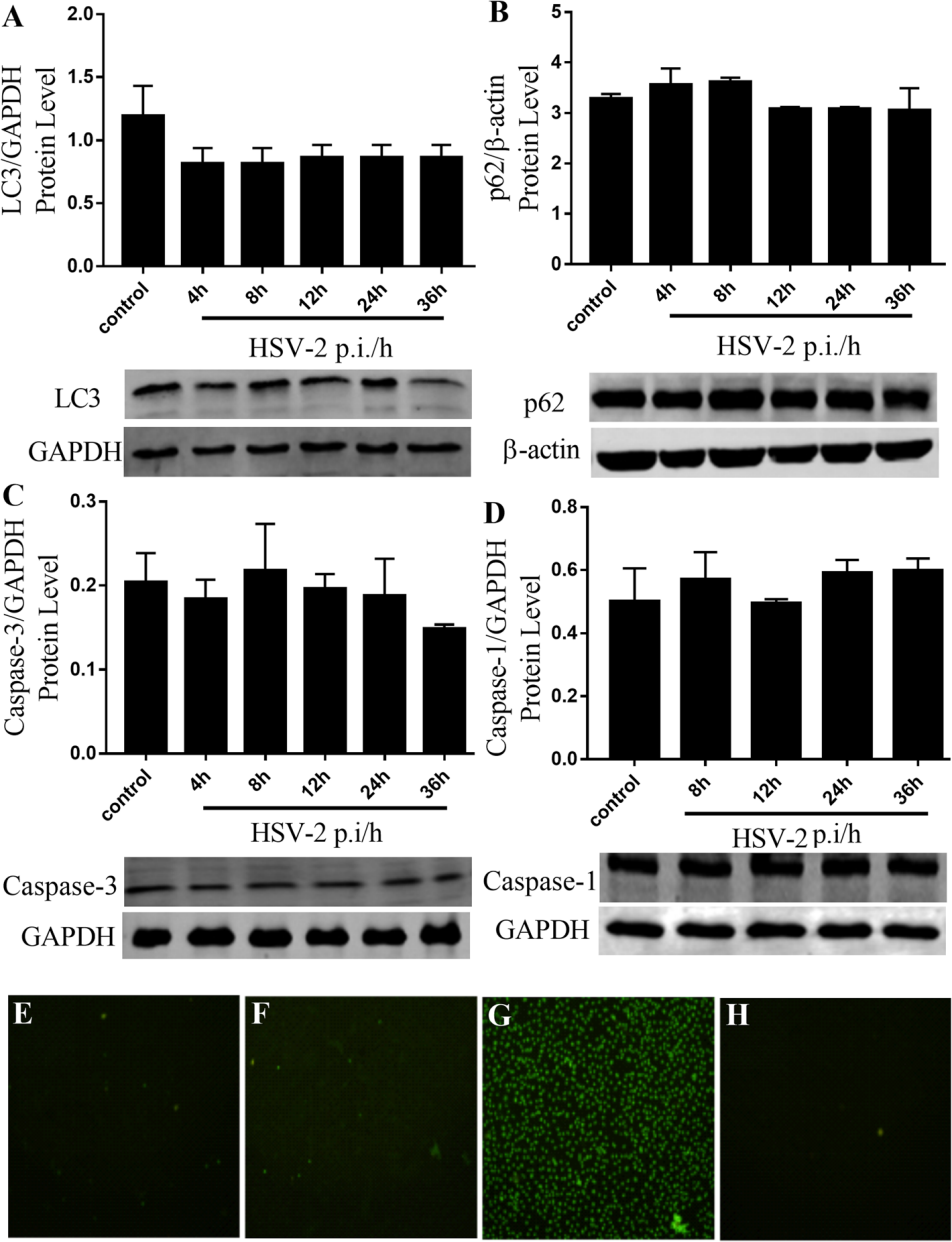
Effects of HSV-2 viral infection on various factors in the four major cell death pathways including autophagy, apoptosis, pyroptosis, and RIP3-mediated programmed cell necrosis pathways. **a** LC3; (**b**) p62; (**c**) caspase-3; (**d**) caspase-1. The TUNEL apoptosis results were shown in (**e**, **f**, **g** and **h**) of the BSC-1 cells. **e** the normal BSC-1 cells, (**f**) the HSV-2 infected cells, (**g**) positive control group, H: negative control group

In this study, it was founded that HSV-2 infection may cause the death of infected cell through RIP3-mediated programmed cell necrosis pathway, but this kind of death had nothing to do with autophagy, apoptosis, and pyroptosis. As shown in Fig. 6, the main relative protein expression levels of autophagy key factors (LC3, p62, Fig. 6a and b), apoptosis-executing factor (Caspase-3, Fig. 6c) and pyroptosis factor (Caspase-1, Fig. 6d) in HSV-2 infected cells were not affected, and the TUNEL apoptosis assay showed that the DNA of the HSV-2 infected cells wasn’t broken compared with the positive control group (Fig. 6e-h).

**Fig. 7:**
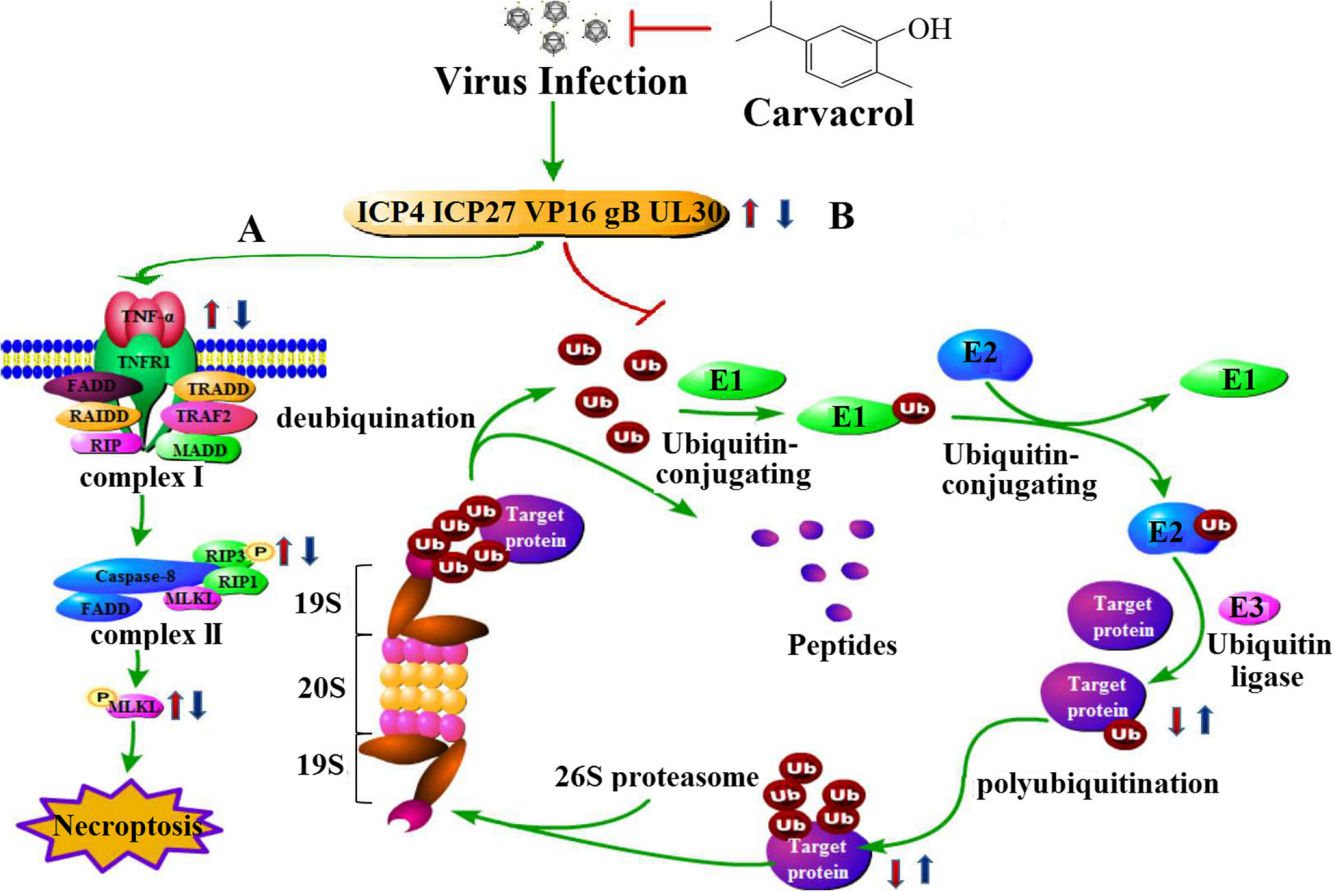
Effect of HSV-2 infection and carvacrol treatment on the RIP3 programmed cell necrosis pathway and the ubiquitin-proteasome degradation pathway on infected cells. **a** the RIP3 programmed cell necrosis pathway; **b** the ubiquitin-proteasome system. Red arrow represent: effect of HSV-2 infection on cells; Blue arrow  represent: effect of carvacrol treatment on HSV-2 infected cells

All mentioned above, carvacrol treatment could induce the RIP3 programmed cell necrosis pathway and the ubiquitin-proteasome degradation pathway on HSV-2 infected cells. As shown in Fig. 7a, HSV-2 infected cells can increase the expression of TNF-α. Subsequently, protein complex I was formed by the binding of TNF-α and its receptor TNF-R1 with the participation of other factors, which in turn initiated the formation of cell death complex II to transmit a death signal. And then, intracellular RIP3-mediated programmed cell necrosis pathway was further initiated by promoting MLKL phosphorylation. But when carvacrol was applied to the infected cells, it may exhibit anti-HSV-2 activity by inhibiting HSV-2-induced TNF-α increased levels, decreasing RIP3 protein activity and weakening MLKL protein expression. The ubiquitin-mediated protein degradation system is that (shown in Fig. 7b) ubiquitin was successively catalyzed by ubiquitin-activating enzyme (E1), ubiquitin-binding enzyme (E2) and ubiquitin ligase (E3), and was bound to the specific lysine of the protein to be degraded. Then, the ubiquitin-target protein is recognized and degraded by the 26S proteasome. Subsequently, the ubiquitin is re-released by the cleavage action of deubiquitinating enzymes and recycledfor the degradation process of the target protein [40]. Our experimental research found HSV-2 infected cells could cause the decrease of intracellular protein ubiquitination level, thereby, hindering protein degradation and destorying the intracellular proteic dynamic balance to cause passive death of cells. When carvacrol is applied to infected cells, it could reverse the ubiquitination decrease level caused by HSV-2 infection and accelerate the degradation of the target protein. So it is speculated that the antiviral activity of carvacrol on infected cells may be related to the ubiquitin-proteasome degradation pathway, however, the specific mechanism that how to influence this system still needs further research and explanation in the future.

In our research, antiviral mechanism of carvacrol on HSV-2 infectivity through inhibition of RIP3-mediated programmed cell necrosis pathway and ubiquitin-proteasome system in BSC-1 cells was study detailedly in vitro the cell line model. In the general population, adult seropositivity rates approach 20–25% for HSV-2. The viruses cause significant morbidity, primarily as genital lesion [41]. At present, the model of HSV-2 infection on primary cell culture model from human reproductive tract tissues has been successfully established [42]. Finally, these finding will have to be further confirmed in the actual primary cells model or animal models.

## Conclusion

In this paper, the antiviral activity and mechanism of carvacrol against herpes simplex virus were investigated in vitro. The anti-HSV activity experiments indicated that the dose-dependent inhibitory activity of carvacrol against HSV-2 was more prominent than that against HSV-1 on the BSC-1 cell model of HSV infection. Both the carvacrol and 2% true carvacrol solution can provide antiviral effects for pre-treatment, treatment and direct inactivation of HSV-2 infection, and carvacrol may exert a good anti-HSV-2 activity in vitro through inhibiting the release process of HSV-2 from intracellular to culture supernatant. At the level of virus replication, carvacrol can significantly inhibit viral replication within 6 ~ 12 h, and inhibit the transcriptional gene and protein expressions levels of ICP4, ICP27, VP16, and gB. Besides, it also can inhibit the formation of the subunit on the DNA polymerase - the UL30 encoding protein, so it may exert antiviral activity by inhibiting the HSV-2 proliferation process. When HSV-2 infection induces multiple cell fusion to form polynuclear giant cells, HSV-2 can initiate intracellular RIP3-mediated programmed cell necrosis pathway by inducing intracellular TNF-α expression, activating RIP3 protein and promoting MLKL expression. When carvacrol was applied on infected cells, it may exhibit anti-HSV-2 activity by inhibiting HSV-2-induced TNF-α increased levels, decreasing RIP3 protein activity and weakening MLKL protein expression. Besides, carvacrol also may exhibit anti-HSV-2 activity by reversing the ubiquitination decrease level caused by HSV-2 infection on the ubiquitin-proteasome system.

## Supplementary Information

**Additional file 1 Figure S1.** Immunofluorescence staining results of ICP27 and gB proteins in BSC-1 cells infected with HSV-2 and in infected cells treated with carvacrol. (A) ICP27; (B) gB.(1. HSV-2; 2. HSV-2 + 0.125 mmol/L carvacrol; 3. HSV-2 + 0.5 mmol/L carvacrol. PCR electrophoresis and melting curves analysis of each gene in HSV-2 infected cells. (C) PCR electrophoresis of each gene in HSV-2 infected cells; D, E, F, G and H represented melting curves analysis of each gene in HSV-2 infected cells (D. ICP4; E. ICP27; F. VP16; G. gB; H. GAPDH).

## Data Availability

The working datasets for statistical analysis in the current study are available from the first author and corresponding author on reasonable request.

## Abbreviations

HSV-2: Herpes simplex virus-2
ACV: Acyclovir
DMEM: Dulbecco-modified eagle’s medium
MOI: Multiplicity of infection
FBS: Fetal bovine serum
IC_50_: Half-maximal inhibitory concentration
EC_50_: 50% effective concentration
TI: Therapeutic indexes
BSA: Bovine serum albumin
PAGE: Polyacrylamide gel electrophoresis
PVDF: Polyvinylidene difluoride
DAPI: 4′,6-diamidino-2-phenylindole
IE: Immediate early
gB: Glycoprotein B
Q-PCR: Quantitative PCR
Ub: Ubiquitination
MRC-5: Human embryonic lung fibroblasts
HEC-1-A: Endometrial adenocarcinoma cells
Hela: Human cervical cancer cells
A549: Human lung cancer cells
Vero: African green monkey kidney cells
BHK: Milk hamster kidney cells
E1: Ubiquitin-activating enzyme
E2: Ubiquitin-binding enzyme
E: Ubiquitin ligase

## References

[1] Yang ZQ, Yu H (2000). Clinical virology.

[2] Drumm CM, Caufield MC, DeKlotz CM (2018). Intrauterine herpes simplex virus infection presenting as a Zosteriform eruption in a newborn. AJP Rep.

[3] Goettsche LS, Wanat KA (2017). Undisturbed characteristic herpes simplex virus 2 outbreak. Dermatol Online.

[4] Paz-Bailey G, Ramaswamy M, Hawkes SJ (2007). Herpes simplex virus type 2: epidemiology and management options in developing countries. Sex Transm Infect.

[5] Smith JS, Robinson NJ (2002). Age-specific prevalence of infection with herpes simplex virus types 2 and 1: a global review. J Infect Dis.

[6] Kimberlin DW (2005). Herpes simplex virus infections in neonates and early childhood. Semin Pediatr Infect Dis.

[7] Farooq AV, Shukla D (2012). Herpes simplex epithelial and stromal keratitis: an epidemiologic update. Surv Ophthalmol.

[8] Li J, Peng F (2012). Research progress of traditional Chinese medicine on anti-herpes simplex virus. Med Recapitulate.

[9] Fiele HJ (2001). Herpes simplex antiviral drug resistance-current trends and future prospects. J Clin Virol.

[10] Piret J, Drouot E, Boivin G (2014). Antiviral drug resistance in herpes viruses. Rev Med Virol.

[11] Ventola CL (2011). The drug shortage crisis in the United States: causes, impact, and management strategies. P T.

[12] Ayse GB, Abdurrahim K, Eray MG (2018). Effects of carvacrol on human ibroblast (WS-1) and gastric adenocarcinoma (AGS) cells in vitro and on Wistar rats in vivo. Mol Cell Biochem.

[13] Sanchez C, Aznar R, Sanchez G (2015). The effect of carvacrol on enteric viruses. Int J Food Microbiol.

[14] Gilling DH, Kitajima M, Torrey JR (2014). Antiviral efficacy and mechanisms of action of oregano essential oil and its primary component carvacrol against murine norovirus. J Appl Microbiol.

[15] Pilau MR, Alves SH, Weiblen R (2011). Antiviral activity of the Lippia graveolens (Mexican oregano) essential oil and its main compound carvacrol against human and animal viruses. Braz J Microbiol.

[16] Miladi H, Zmantar T, Kouidhi B (2017). Synergistic effect of eugenol, carvacrol, thymol, p-cymene and γ-terpinene on inhibition of drug resistance and biofilm formation of oral bacteria. Microb Pathog.

[17] Han X, Parker TL (2017). Anti-inflammatory, tissue remodeling, immunomodulatory, and anticancer activities of oregano (*Origanum vulgare*) essential oil in a human skin disease mode. Biochim Open.

[18] Ozturk H, Cetinkaya A, Duzcu SE (2018). Carvacrol attenuates histopathogic and functional impairments induced by bilateral renal ischemia/reperfusion in rats. Biomed Pharmacother.

[19] Vahideh G, Azam A, Omid R, Amir HM, Mohammad HB (2018). Safety and tolerability of carvacrol in healthy subjects: a phase I clinical study. Drug Chem Toxicol.

[20] Azam A, Mohammad RK, Mohammad HB (2018). Possible therapeutic effect of carvacrol on asthmatic patients: a randomized, double blind, placebo - controlled, phase II clinical trial. Phytother Res.

[21] Sharifi-Rad J, Salehi B, Schnitzler P, Ayatollahi S, Kobarfard F, Fathi M (2017). Susceptibility of herpes simplex virus type 1 to monoterpenes thymol, carvacrol, p-cymene and essential oils of *Sinapis arvensis* L., Lallemantia royleana Benth. And Pulicaria vulgaris Gaertn. Cell Mol Biol (Noisy-le-Grand, France).

[22] Toujani MM, Rittà M, Civra A, Genovese S, Epifano F, Ghram A (2018). Inhibition of HSV-2 infection by pure compounds from Thymus capitatus extract in vitro. Phytother Res.

[23] Lai WL, Chuang HS, Lee MH (2012). Inhibition of herpes simplex virus type 1 by thymol-related monoterpenoids. Planta Med.

[24] Munetaka I, Fumio O, Kazumi S, Tomoyuki H, Keiji S, Masami W (1999). A water soluble Tetrazolium salt useful for colorimetric cell viability assay. Anal Commun.

[25] Su CT, Hsu TA, Hsieh HP, Lin PH, Chen TC, Kao CL (2008). Anti-HSV activity of digitoxin and its possible mechanisms. Antivir Res.

[26] Zhen H, Fang F, Ye DY (2006). Experimental study on the action of allitridin against human cytomegalovirus in vitro: inhibitory effects on immediate-early genes. Antivir Res.

[27] You Y, Cheng AC, Wang MS (2017). The suppression of apoptosis by α-herpesvirus. Cell Death Dis.

[28] Sacks WR, Greene CC, Aschman DP (1985). Herpes simplex virus type 1 ICP27 is an essential regulatory protein. J Virol.

[29] Mingo RM, Han J, Newcomb WW, Brown JC (2012). Replication of herpes simplex virus: egress of progeny virus at specialized cell membrane sites. J Virol.

[30] Granzow H, Klupp BG, Fuchs W, Veits J, Osterrieder N, Mettenleiter TC (2001). Egress of alphaherpesviruses: comparative ultrastructural study. J Virol.

[31] Owen DJ, Crump CM, Graham SC (2015). Tegument assembly and secondary envelopment of Alphaherpesviruses. Viruses.

[32] Wenwen D, Yu W, Jinpeng B, Jingyu W, Shuai W, Wei K (2018). Antiviral effect of Retro-2.1 against herpes simplex virus type 2 in vitro. J Microbiol Biotechnol.

[33] Qiu M, Wu ZW (2014). The mechanism of anti-herpes simplex virus (HSV) effect of zinc Ionophore and preliminary study of the relationship between ubiquitin-proteasome pathway and HSV replication [PhD thesis].

[34] Jenkins FJ, Turner SL (1996). Herpes simplex virus: a tool for neuroscientists. Front Biosci.

[35] Uprichard SL, Knipe DM (1996). Herpes simplex ICP27 mutant viruses exhibit reduced expression of specific DNA replication genes. J Virol.

[36] Smiley JR (2004). Herpes simplex virus virion host shutoff protein: immune evasion mediated by a viral RNase?. J Virol.

[37] Weller SK, Coen DM (2012). Herpes simplex viruses: mechanisms of DNA replication. Cold Spring Harb Perspect Biol.

[38] Xiaoliang Y, Sudan H (2016). The interplay between human herpes simplex virus infection and the apoptosis and necroptosis cell death pathways. Viol J.

[39] Kennedy PGE (2015). Viruses, apoptosis, and neuroinflammation - a double-edged sword. J Neuro-Oncol.

[40] Glickman MH, Ciechanover A (2002). The ubiquitin-proteasome proteolytic pathy: destruction for the sake of construction. Physiol Rev.

[41] Kollias CM, Huneke RB, Wigdahl B, Jennings SR (2015). Animal models of herpes simplex virus immunity and pathogenesis. J Neuro-Oncol.

[42] MacDonald EM, Savoy A, Gillgrass A, Fernandez S, Smieja M, Rosenthal KL (2007). Susceptibility of human female primary genital epithelial cells to herpes simplex virus, type-2 and the effect of TLR3 ligand and sex hormones on infection. Biol Reprod.

