# Supplementary figures and images for: Antiviral mechanism of carvacrol on HSV-2 infectivity through inhibition of RIP3-mediated programmed cell necrosis pathway and ubiquitin-proteasome system in BSC-1 cells
Source: BMC Infect Dis. 2020 Nov 11;20:832. doi: 10.1186/s12879-020-05556-9 (PMC7661259; doi:10.1186/s12879-020-05556-9)

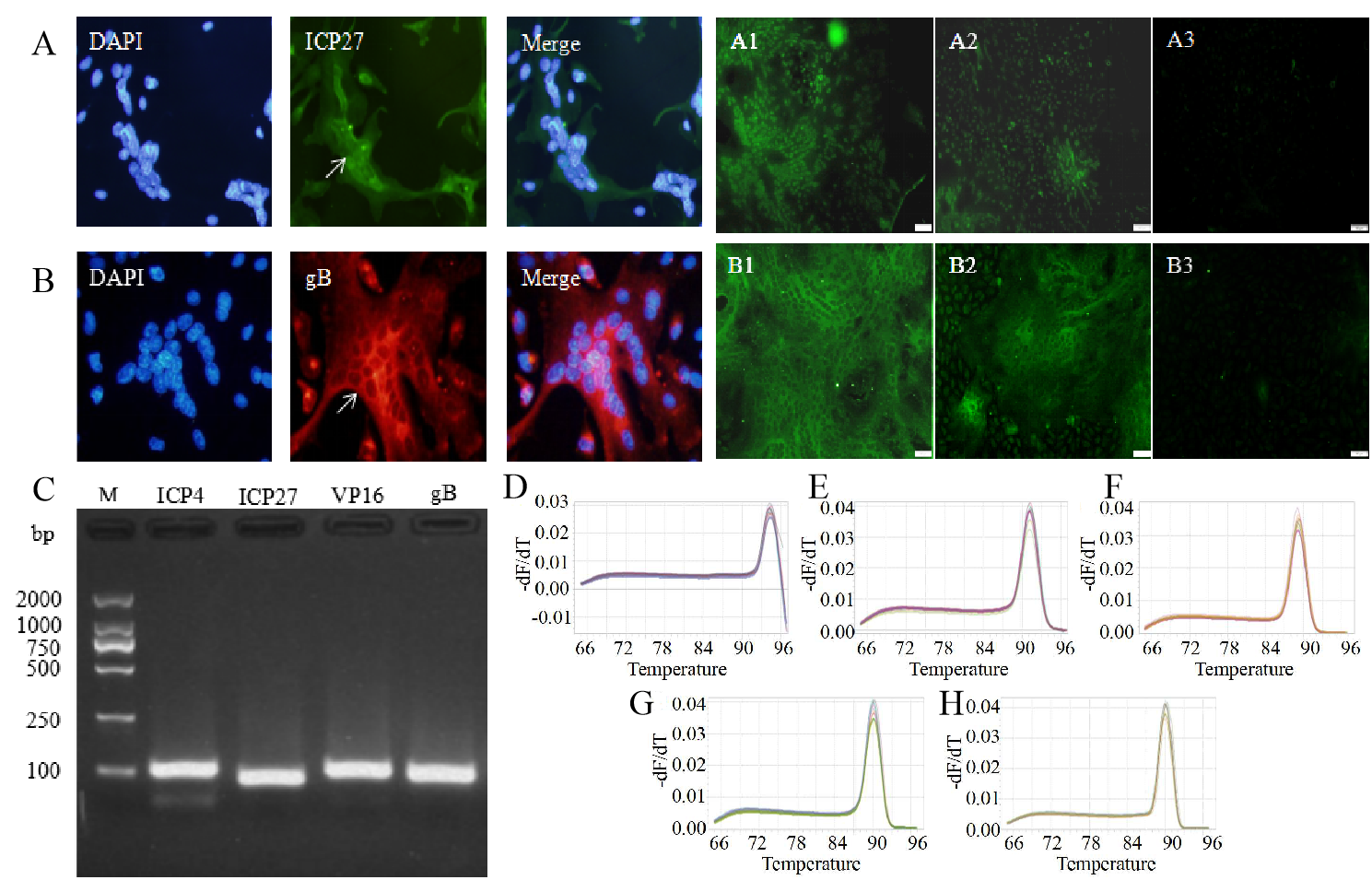

Supplement: Supplementary file 1 — Additional file 1 Figure S1. Immunofluorescence staining results of ICP27 and gB proteins in BSC-1 cells infected with HSV-2 and in infected cells treated with carvacrol. (A) ICP27; (B) gB.(1. HSV-2; 2. HSV-2 + 0.125 mmol/L carvacrol; 3. HSV-2 + 0.5 mmol/L carvacrol. PCR electrophoresis and melting curves analysis of each gene in HSV-2 infected cells. (C) PCR electrophoresis of each gene in HSV-2 infected cells; D, E, F, G and H represented melting curves analysis of each gene in HSV-2 infected cells (D. ICP4; E. ICP27; F. VP16; G. gB; H. GAPDH). [file 12879_2020_5556_MOESM1_ESM.tif]
